# Analysis of L-leucine amino acid transporter species activity and gene expression by human blood brain barrier hCMEC/D3 model reveal potential LAT1, LAT4, B^0^AT2 and y^+^LAT1 functional cooperation

**DOI:** 10.1177/0271678X211039593

**Published:** 2021-08-24

**Authors:** Mehdi Taslimifar, Martin Faltys, Vartan Kurtcuoglu, François Verrey, Victoria Makrides

**Affiliations:** 1 The Interface Group, Institute of Physiology, University of Zürich, Zürich, Switzerland; 2 Epithelial Transport Group, Institute of Physiology, University of Zürich, Zürich, Switzerland; 3 Department of Intensive Care Medicine, University Hospital, University of Bern, Bern, Switzerland; 4 National Center of Competence in Research, Kidney CH, Switzerland; 5 EIC BioMedical Labs, Norwood, MA, USA

**Keywords:** Blood-brain barrier, computational model, insulin-like growth factor 1, hCMEC/D3, solute carrier amino acid transporters

## Abstract

In the CNS, amino acid (AA) neurotransmitters and neurotransmitter precursors are subject to tight homeostatic control mediated by blood-brain barrier (BBB) solute carrier amino acid transporters (AATs). Since the BBB is composed of multiple closely apposed cell types and opportunities for human *in vivo* studies are limited, we used *in vitro* and computational approaches to investigate human BBB AAT activity and regulation. Quantitative real-time PCR (qPCR) of the human BBB endothelial cell model hCMEC/D3 (D3) was used to determine expression of selected AAT, tight junction (TJ), and signal transduction (ST) genes under various culture conditions. L-leucine uptake data were interrogated with a computational model developed by our group for calculating AAT activity in complex cell cultures. This approach is potentially applicable to *in vitro* cell culture drug studies where multiple “receptors” may mediate observed responses. Of 7 Leu AAT genes expressed by D3 only the activity of SLC7A5-SLC3A2/LAT1-4F2HC (LAT1), SLC43A2/LAT4 (LAT4) and sodium-dependent AATs, SLC6A15/B^0^AT2 (B^0^AT2), and SLC7A7/y^+^LAT1 (y^+^LAT1) were calculated to be required for Leu uptake. Therefore, D3 Leu transport may be mediated by a potentially physiologically relevant functional cooperation between the known BBB AAT, LAT1 and obligatory exchange (y^+^LAT1), facilitative diffusion (LAT4), and sodium symporter (B^0^AT2) transporters.

## Introduction

In the brain, amino acids (AA) not only participate in protein synthesis and other metabolic pathways, many are also neurotransmitters or neurotransmitter precursors. Therefore, to maintain healthy brain physiology, CNS AA concentrations must be robustly regulated. Tight homeostatic control of AAs depends, in part, on the activity of solute carrier family (SLC) AA transporters (AATs) localized on the blood-brain barrier (BBB) microvascular endothelium.^1–3^ Since the BBB relies on multiple closely apposed cell types, investigating the physiological roles of endothelial transporters *in vivo* is technically difficult even in animal models. In addition, human studies are generally restricted to minimally invasive methods such as MRI, PET or CT scans. While, for example, PET generates data related to solute transport, the circumstances and types of solutes probed are often restricted to diagnostic studies using a limited set of probes. Consequently, *in vitro* approaches have been utilized to probe BBB endothelial transport, including studies using brain microvascular endothelial and other types of cell culture models. Obtaining human material can be limiting and, therefore, many of the commonly used brain microvascular endothelial cell culture models are from non-human progenitor cells. These models, while forming a relatively tight barrier, also potentially introduce species-specific anomalies. The brain microvascular endothelial hCMEC/D3 (D3) cell line, which demonstrates many salient features of BBB endothelial cells, is one of a few immortalized human derived brain microvascular models.^4–8^ Their suitability as a BBB *in vitro* model has been extensively probed. For example, D3 cells have been reported to express a number of proteins including transporters, tight junction and signaling molecules that are consistent with their origin from brain endothelium.^8–11^ In addition, D3 cells have been utilized in numerous studies investigating brain endothelial physiological and pathological functions.^5,7,8,12,13^

In this study we used D3 to parse the potential roles of human BBB endothelial AATs that participate in generating, maintaining and regulating the steep AA concentration gradient measured from plasma to brain interstitial fluid (ISF).^1–3,13^ Amino acid transporters function by three general mechanisms: exchange of one AA for another substrate (antiporter); coupled transport of two or more substrates in the same direction (symporter); and transport across the membrane driven by the AA concentration gradient (facilitative diffusion). Additionally, transport may be dependent or independent of specific ions such as sodium. Therefore, AATs can be categorized as sodium-dependent (Na^+^-dep) or sodium-independent (Na^+^-indep) transporters. Cell culture models recapitulate much of the complexity of *in vivo* AA transport in which multiple AATs with distinct kinetics and potentially overlapping substrate specificities mediate transport of a given substrate. This complexity obscures the roles of individual AATs. Previously, we developed and validated a mathematical model to quantitatively parse individual activities in the context of complex cellular environments in which multiple SLCs may mediate transport of a given solute.^14^ Several groups have tested AAT D3 gene and protein expression.^8–11,15^ Here, we additionally calculated contributions to transport by specific D3 AAT species; data which may shed light on physiological roles of brain microvascular endothelial AATs in regulating CNS AA homeostasis. To carry out this analysis, we measured transport of the essential branched chain mTOR activator L-leucine (Leu) by D3 cells. Among AAs with measurable brain ISF levels,^1^ Leu has the highest blood to brain influx rate.^16–18^ While not neurotransmitters, branched chain AAs, particularly Leu, can serve as a potential amino group donor for neurotransmitter synthesis.^18^ Furthermore, the Large Neutral AA (LNAA) Na^+^-indep anti-porter, LAT1-4F2/SLC7A5-SLC3A2 (LAT1), which transports Leu, is a specific *in vivo* cell marker for BBB endothelium.^1–3,19,20^

Based in part on previously reported microarray data for D3 grown in rich media^15^ the mRNA expression of D3 AAT, tight junction (TJ) and signaling molecules were quantified by RT-qPCR. We confirmed mRNA expression of 7 Leu AATs including LAT1 in D3. A computational analysis based on known AAT Leu affinities of the potentially expressed plasma membrane AATs was carried out using Leu uptake data measured under conditions that allowed application of the MM equation. Calculations indicated activity of two Na^+^-indep (LAT1 and LAT4) and two Na^+^-dep (B^0^AT2 and y^+^LAT1) AATs were sufficient to account for D3 Leu transport. Cell culture models are known to be influenced by a number of extrinsic factors such as length of culture and media composition including growth factor supplementation. Therefore, to determine whether D3 Leu transport might be affected by these factors we investigated their potential effects on gene expression and activity.

## Materials and methods

### Cell culture

Human brain endothelial cells (hCMEC/D3, short D3) were obtained from the lab of Pierre-Olivier Couraud under license from Institut national de la santé et de la recherche médicale (Inserm, Paris, France). For mRNA and uptake studies, passage 31 to 37 D3 cells were cultured in Transwell™ plates (Costar, Corning Inc., Corning NY, USA) on collagen coated (150 µg/ml rat type I collagen, Cultrex, R&D Systems, Abingdon, United Kingdom) polyester membrane filter inserts with 0.4 µm pore size. The experimental protocols, which varied by the specific media used, passage number density of cells plated, length of total culture and sampling day(s), are indicated in the results section and figure legends. Cells were cultured in rich media (RM) consisting of EBM-2 medium supplemented with 0.8 mg/ml sodium bicarbonate, 2.5% fetal bovine serum (FBS), ¼th volume of Cambrex Bullet kit supplements, or minimal media (DM) consisting of EBM-2 medium supplemented with 0.8 mg/ml sodium bicarbonate, 2.5% FBS, 1.4 µM hydrocortisone, 5 µg/mL ascorbic acid, 1 ng/mL basic FGF (bFGF) with or without insulin-like growth factor 1 (IGF1) supplementation (¼th volume of Cambrex Bullet kit IGF1). Unless otherwise stated, all media and supplements were supplied by Lonza, Walderswill MD, USA. The cells were cultured at 37 °C, 5% CO_2_, saturated humidity. Cell culture medium was exchanged every 1–3 days.

### Quantitative real-time PCR analyses

#### RNA purification and cDNA preparation

cDNA was produced from total RNA extracted from homogenized cell lysates using RNAeasyplus™ or using RNAeasy™ kits with an additional 5 min incubation step in RW1 buffer and on column DNAase digestion as described in the manufacturer’s manual (Qiagen AG, Hombrechtikon, Switzerland). RNA concentrations were determined using the NanoDrop ND 1000 (NanoDrop Technologies, Wilmington, DE, USA) at the University of Zurich or with the NanoDrop One (Thermo Fisher Scientific, Waltham, MA, USA) at Syd Labs (Syd Labs, Natick, MA). Purified RNA concentrations varied from 50–450 ng/µl. RNA was reverse transcribed to cDNA (+RT) either with the GenAmp RNA PCR Kit (Applied Biosystems) following the manufacturer’s protocol using a final RNA concentration of 20 ng/µL or with Syd Labs First Strand cDNA Synthesis Kit at a final concentration of 45 ng/µL. For the negative control cDNAs (-RT) reactions were carried out with H_2_O replacing the reverse transcriptase enzyme. No further purification of cDNA was performed. Hypoxanthine phosphoribosyl transferase 1 (HPRT, housekeeping gene in purine salvage pathway) was used as the reference for normalizing expression among cells grown in various conditions. cDNA quality was tested using HPRT primers in qPCR reactions for all +RT cDNA samples and -RT cDNA negative controls (Table S1). Control ±RT cDNA was prepared from human total brain or total kidney RNA (Clontech, Invitrogen).

#### Primers

Primers were designed for a selected panel of human transporter, regulatory, and TJ protein genes (Table S1). Initial gene selection was made partially on the basis of *in vivo* gene expression studies previously performed on rapidly isolated and highly purified mouse brain microvascular endothelial RNA^2^ and on previously reported D3 microarray data.^15^ Primer design was carried out using the Primer Express 3™ software with a targeted amplicon length of 100-200 base pairs. All primers were validated by SYBR Green™ quantitative PCR (qPCR) in three ways. First, primer specificity for each gene was tested using control human brain or kidney ±RT cDNA or using mixed and diluted aliquots of D3 cDNA samples (Syd Labs) or water-only (no template) qPCR reactions and confirmed by DNA electrophoresis. Second, the optimum primer concentration was determined by titration of forward and reverse primers. Finally, primer efficiency (E), defined as E = 10^(-1/m)-^1, where m is the slope of the linear regression of the CT values vs. log ng/reaction +RT cDNA, was assessed by titration vs. ±RT cDNA concentration. Primers with E ≥ 1.6 were used for further experiments.

#### qPCR reactions

Gene expression was determined as previously described using Real-time qPCR assays^2^ either at the University of Zürich, Institute of Physiology using SYBR Green™ qPCR (Sigma) according to the manufacturer’s instructions or at Syd Labs using SYBR Green Universal qPCR Master Mix (Syd Labs). All qPCR reactions were performed as technical triplicates using the Fast Real-Time PCR System 7500 (Applied Biosystems) at the University of Zurich or the Bio-Rad CFXconnect (Bio-Rad) at Syd Labs. Relative expression values for D3 cells grown in rich media were calculated by the comparative ΔC_T_ method relative to HPRT expression (relative expression = 2^−ΔC_T_^, ΔC_T_ value = average C_T_ value of target-average C_T_ value of endogenous HPRT reference). Analysis of transporter gene relative expressions of cells grown in minimal media (DM) ± IGF1 were analyzed using the ΔΔC_T_ method relative to expression in D3 cultures at day 2 in DM ((relative expression = 2^−ΔΔC_T_^, ΔΔC_T_ value = (average C_T_ value of target in target sample-average C_T_ value of HPRT in target sample) – (average C_T_ value of target gene in control sample-average C_T_ value of HPRT in control sample). Data are available upon reasonable request.

#### Statistics

All qPCR results are expressed as the mean ± SD of the relative quantification vs. HPRT expression. Data subject to statistical analysis were tested for normality using Shapiro-Wilk and/or Kolmogorov-Smirnov with Dallal-Wilkinson Liffliefor P value using Prism (ver 5 and/or 9; GraphPad, San Diego, CA, USA). One-way Anova with the Tukey or Bonferroni posttests performed using Prism 5 were used to analyze differences between results. P-values below 0.05 were considered to indicate statistically significant differences.

### Amino acid uptakes assays

Amino acid uptake assays were performed on passage 31-37 confluent D3 cells grown on Corning Transwell™ filters in media as indicated in results and figure legends. Assays were carried out as previously described with some modifications.^21^ Briefly, cells were washed 3 times and incubated for 10 min at 37 °C in ± sodium buffers as indicated. Both sodium (+Na^+^) containing buffers prepared with 118 mM sodium and sodium free (Na^+^free) buffers prepared with equal molar N-methyl-D-glucamine (NMDG) contained 10 mM Tris pH 7.4, 1.25 mM CaCl_2_, 4.7 mM KCl, 1.2 mM MgSO_4_. Uptake buffers were prepared by supplementation of +Na^+^ or Na^+^free solutions with various concentrations of L-Leucine (Leu) (as indicated in text and figure legends) and 2 µCi/ml of ^3^H-labeled L-Leucine (Perkin Elmer, Shelton, CT, USA) as AA tracer. Uptake solutions were applied to apical and basolateral Transwell™ chambers. After 1 min incubation at 37 °C, uptakes were stopped by removing uptake solutions and washing 6 times with respective ice-cold buffers, filters were excised and shaken overnight in scintillation fluid at room temperature. Radioactivity was measured at the Institute of Physiology (University of Zürich, Zurich, Switzerland) or by Matt Mahowald at the Nuclear Reactor Laboratory, Massachusetts Institute of Technology (MIT, Cambridge, MA, USA) using a Packard Tri-Carb 2900TR liquid scintillation analyzer (PerkinElmer).

### Determination amino acid transporter species uptake rates

Data (calculated as pmol/(min^.^cm^2^) of filter area and normalized to uptake rates for 300 µM Leu) were tested for normality using Shapiro-Wilk and/or Kolmogorov-Smirnov with Dallal-Wilkinson Liffliefor P value using Prism. The Michealis-Menten (MM) equation was fitted to the AA uptake data by nonlinear regression to calculate transport kinetic parameters, namely maximum transport rate (*V_max_*) and MM binding constant (*K_m_*) using Prism. As previously described,^14^ analysis of D3 uptake responses for a range of Leu concentrations in ± Na^+^ buffers were used to calculate *V_max_* for each individual AAT species (*V_max,i_)* presumed present from microarray data^15^ and confirmed present by quantitative real-time polymerase chain data (qPCR). These individual maximum transport rates will be referred to as *V_max,I_*, where *i* designates the ith AAT species considered. *V_max,i_* was determined by global fitting in OriginPro (versions 2020, 2021; Origin Lab, Northampton, MA, USA) using least squares optimization over all data, assuming that the maximum uptake rate is equal for all uptake buffers^22,23^ (Table S2). Specifically parameter values were modified iteratively to minimize the reduced Chi-Square (Chi^2^) value, which is the weighted sum of the squared differences (RSS) between model output and measured data divided by the degrees of freedom (DOF). The adjusted R^2^ value, for which the coefficient of determination R^2^ is modified to take into account the model DOF relative to the number of measured data points, was used to assess closeness of fit. Values closer to 1 indicate better fit of the model to the data. Based on the estimated *V_max,i_* and the *K_m_* values for each human AAT (*K_m,i_*) as reported in the literature (Table 1), the uptake rate by each described species of AAT was calculated for various concentrations of extracellular Leu in +Na^+^ and in Na^+^free uptake buffers (Figure 2, Table S2). Data are available upon reasonable request.

**Table 1.** Blood-brain barrier transporters model input parameters (*V_max_* and *K_m_*) for L-leucine.

	Symporter	Antiporter						Uniporter	
SLC no.	SLC6A15	SLC7A5	SLC7A9		SLC7A7	SLC7A6	SLC1A5	SLC43A2	
Alias	B^0^AT2	LAT1	b^0+^AT		y^+^LAT1	y^+^LAT2	ASCT2	LAT4	
Accessory Protein	NA	4F2HCSLC3A2	rBATSLC3A1		4F2HCSLC3A2	4F2HCSLC3A2	NA	NA	
								*V*_max_^1^	*V*_max_^2^
*V_max_* (nmol/h)	NA	0.162		NA	0.38	1.60	1.01	0.024	3.52
*K_m_* (mM)								*K*_m_^1^	*K*_m_^2^
	0.160	0.032		1.1	0.0317	0.236	0.367	0.103	3.73
Sodium dependent	yes	no		no	yes	yes	yes	no	

Note: References for kinetic values for model input parameters for: hB^0^AT2 *K_m_*^39^; hLAT1 *V_max_*^56^ and *K_m_*^57^; mb^0+^AT *K_m_*^58^; hy^+^LAT1 *K_m_*^47^ and *V_max_*^47^; hy^+^LAT2 *K_m_*^48^ and *V_ma_*_x_^48^; mASCT2 *K_m_*^59^ and *V_max_*^59^; hLAT4 *K_m_*^24^ and *V_max_*.^24^ If *V_max_* rates were originally not reported in units of nmol/h, they were recalculated converted. NA: not available.

## Results

### hCMEC/D3 cells express high levels of LAT1, LAT4 and B^0^AT2 amino acid transporter transcripts

To understand the amino acid transport functions of the human BBB endothelial cell model hCMEC/D3, we first measured the mRNA expression of several transporter, TJ or cell signaling genes reported present by microarray in such D3 cells cultured in a rich media (RM).^15^ For these studies, target gene expression was quantified relative to the housekeeping gene hypoxanthine phosphoribosyl transferase (HPRT, mean threshold cycle (C_T_) for D3 grown in RM was 18.85 ± 0.49 (mean ± SEM)).

**Figure 1.**
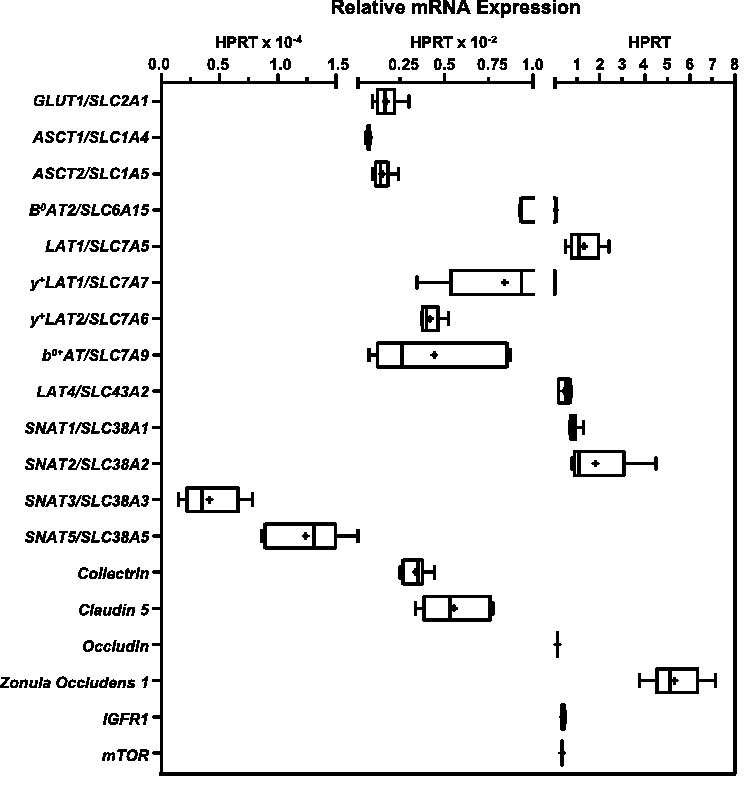
Gene expression of transporters, tight junction proteins and selected signaling molecules by the human endothelial blood-brain barrier (BBB) endothelial *in vitro* cell culture model, hCMEC/D3 (D3). The gene expression of D3 (p31-34) cells cultured for 2–6 days in rich media on Transwell™ membranes was assayed by SYBR Green real-time quantitative PCR (qPCR). Gene expression was normalized to expression of the housekeeping gene *HPRT1* (2^−ΔC_T_^) and is shown as box (25 to 75 percentiles) and whisker plots of minimum to maximum values with the median indicated by a line and mean marked (+). n = 5–24 biological samples (3 technical replicates per sample) assayed from 2–4 independent experiments per gene. Primer sequences and gene accession (NM_) numbers are reported in Table S1.

Figure 1 shows the D3 qPCR quantification of mRNAs for the solute carrier (SLC) transporters for glucose (*GLUT1/SLC2A1 (GLUT1*)) or amino acid transporters *ASCT1/SLC1A4 (ASCT1*)*, ASCT2/SLC1A5* (*ASCT2*)*, B^0^AT2/SLC6A15* (*B^0^AT2*)*, LAT1/SLC7A5* (*LAT1*)*, y^+^LAT1/SLC7A7* (*y^+^LAT1*)*, y^+^LAT2/SLC7A6* (*y^+^LAT2), b*0*^,+^AT/SLC7A9* (*b^0,+^AT*)*, LAT4/SLC43A2* (*LAT4*)*, SNAT1/SLC38A1* (*SNAT1*)*, SNAT2/SLC38A2* (*SNAT2*)*, SNAT3/SLC38A3* (*SNAT3*)*, SNAT5/SLC38A5* (*SNAT5*), the transporter associated-protein *Collectrin/CLTRN (COLL*), the TJ proteins genes *Claudin-5/CLDN5, (CLDN5*), *Occludin/OCLDN (OCLDN*), Zonula Occluden-1/*ZO1* (ZO1), as well as the signaling molecules *mammalian target of rapamycin*/*mTOR* (mTOR)*, Insulin-like growth factor receptor 1*/*IGFR1* (IGFR1). Gene expressions clustered around three general levels. Several AAT (*B^0^AT2, LAT1, SNAT1, SNAT2* and *LAT4*) and signaling molecules (*IGFR1*, *mTOR*) and TJ protein genes (*ZO1, OCLDN*) demonstrated expression levels from 10% to comparable or greater (*ZO1*) than *HPRT*. The majority of the SLC mRNAs including the endothelial cell marker, *GLUT1,* and the AATs, *ASCT1, ASCT2, y^+^LAT1, y^+^LAT2,* and *b^0+^AT*, as well as the associated protein COLL and the TJ protein CLND5 were expressed from 100 to 1000 times lower than HPRT. The mRNAs for *SNAT3* with known brain microvascular endothelial protein expression and membrane localization, and for *SNAT5,* whose mRNA but not protein has been detected expressed by BBB endothelium *in vivo*^2,3^ were detected in D3 at approximately 10^−4^ or lower levels than HPRT.

### D3 cells exhibit both sodium dependent and sodium independent L-leucine uptake

**Figure 2.**
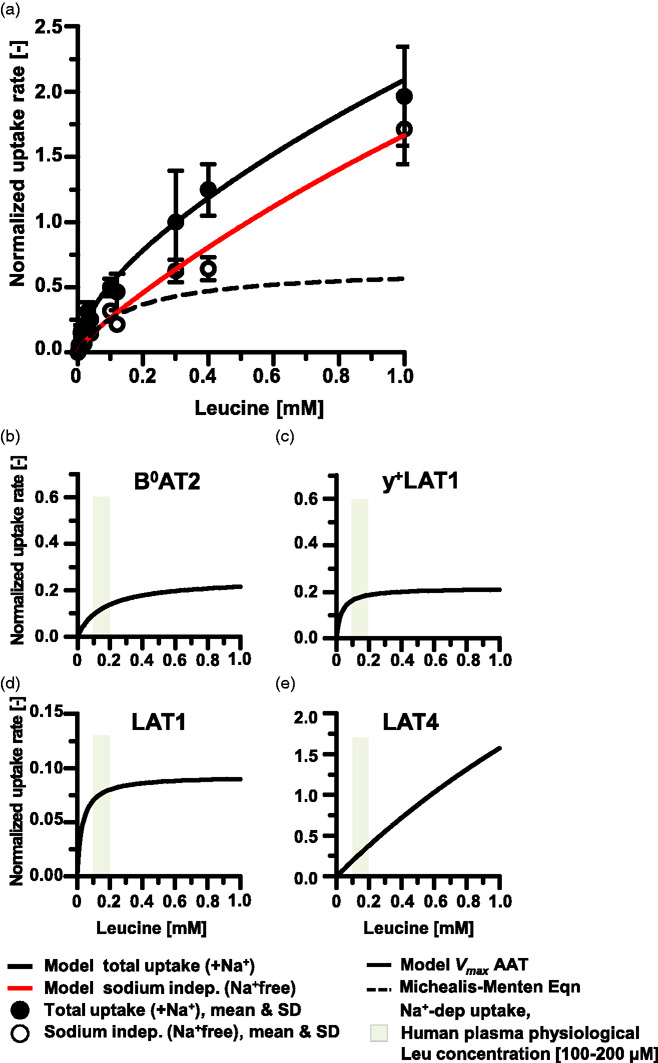
hCMEC/D3 L-leucine plasma membrane amino acid transporter activity. L-leucine (Leu) uptake rates by p34–36 hCMEC/D3 (D3) cells cultured in rich media for 7–15 days were determined in solutions containing sodium (+Na^+^) corresponding to Total Uptake (filled circles) by all AATs and in sodium-free solutions (Na^+^free) corresponding to uptake by Na^+^-indep (open circles) AATs. Leu (10–1000 µM Leu) uptake rates measured in units of pmol/(min.cm^2^) were normalized to rates for 300 µM Leu. (a) The mean and standard deviation (SD) for 5–12 filters per concentration Leu assayed from 2–4 independent experiments are shown. Model calculated regressions for Total and Na^+^-indep uptake rates are plotted with solid lines. Na^+^-dep uptake rate calculated as Total uptake minus Na^+^-indep uptake with the Michaelis-Menten equation-derived fit to the data is shown plotted with a dotted line. Panels b–e show the calculated contributions towards Leu uptake carried out by the identified AATs as reported in Table S2. The uptake rates by individual AAT species were simulated for 0–1 mM Leu and graphed in panels as: (b) B^0^AT2, (c) y^+^LAT1, (d) LAT1, and (e) LAT4_low_.

The amino acid transporter activity by D3 for a range of Leu concentrations (0.01, 4, 10, 12, 20, 30, 50, 40, 100, 120, 300, 400, 1000 µM) was assayed for 1 min in the presence and absence of Na^+^ (Figure S1, Figure 2(a)). The specific subset of Leu concentrations assayed varied by experiment. Data on uptake rates, V, were reported as pmol/min per cm^2^ of cultured cells normalized to rates at 300 µM. Uptake carried out in the presence of Na^+^, i.e. in +Na^+^ buffers, permits uptake by both Na^+^-dep and Na^+^-indep D3 AATs and is referred to as Total uptake. Whereas uptake carried out the absence of Na+ i.e. in Na^+^free buffers, corresponds to transport by Na^+^-indep AATs alone. It is conventional to determine the uptake carried out by Na^+^-dep AATs by “subtracting” Na^+^-indep uptake from Total Uptake, therefore Na^+^-dep uptake rate was calculated as the difference between measured *V* in these two buffer conditions (Figure 2(a)).

Figure 2(a) shows the mean ± standard deviation of the mean (SD) and the model regression fit for Leu uptake rates ±Na^+^. Under these conditions, the maximum velocity rate (*V_max_*) for the total transport of Leu (0-1mM) derived by fitting the MM equation to the data was 2.99 ± 0.25 pmol/(min.cm^2^). The aggregate *V_ma_*_x_ for the Na^+^-indep vs the Na^+^-dep AATs were calculated as 9.5 ± 5.3 vs 0.65 ± 0.08 pM/(min.cm^2^) and Leu affinities, expressed in terms of the binding constant *K_m_* were calculated as 4.6 ± 2.98 and 0.156 ± 0.048 mM, for Na^+^-indep and Na^+^-dep AATs, respectively (Figure 2(a)).

### LAT1, LAT4, B^0^AT2 and y^+^LAT1activities contribute to L-Leucine transport

The kinetic parameters for the activities of the 7 Leu AATs with detectable D3 mRNA expression by microarray^15^ are listed in Table 1. LAT4 has been shown to display both high and low affinity Leu transport,^24^ resulting in 8 AAT kinetic activities potentially mediating D3 Leu transport.

Based on the normalized experimentally determined mean uptake rates, contributions of individual AAT species to Leu transport by D3 cells were determined computationally as briefly described in the methods section (we refer the reader to a detailed description and validation of the approach in Taslimifar et al.^14^) Figure 2 panels a-e and Table S2 show the calculated Leu (0–1000 µM Leu) maximum transport rates (*V_max,i_*) for 8 AAT (including LAT4_high_ and LAT4_low_) as: B^0^AT2 (Figure 2(b), *V_max,i_ *= 0.2492 ± 15 pmol/min); y^+^LAT1 (Figure 2(c), *V_max,i_ *= 0.2161 ± 0.72 pmol/min); LAT1 (Figure 2(d), *V_max,i_ *= 0.0927 ± 0.39 pmol/min): and LAT4_low_ (Figure 2(e), *V_max,i_ *= 7.448 ± 8.95 pmol/min). No activity was calculated for ASCT2, y^+^LAT2, b^0,+^AT and high affinity LAT4_high_ Leu transport. The corresponding reduced Chi2 and adjusted R^2^ were 10^−9^ and 0.983, respectively. The range of Leu human physiological plasma levels^25^ (98-205 µM) are indicated with a grey band (Figure 2(b) to (e)). Transport rates by AAT calculated for this range indicate nearly equivalent activities of B^0^AT2, y^+^LAT1, LAT1 and LAT4_low_. However, with increasing Leu concentration, LAT4_low_ transport rapidly outstrips activity of the remaining three transporters.

From Table 1 it can be seen that reported Leu affinities of three of the four Na^+^-dep AATs (B^0^AT2, y^+^LAT2, ASCT2) cluster in the range of 160–367 µM. Additionally, the Leu *K_m_* of the fourth Na^+^-dep AAT, y^+^LAT1 (0.0317 mM), and the high affinity Na^+^-indep AATs, LAT1 (0.032 mM) and LAT4_high_ (0.103 mM) are comparable and likewise, *K_m_* of b^0+^AT (1.1 mM) and the low affinity component of LAT4_low_ (3.7 mM) are of the same order of magnitude. From qPCR data, *B^0^AT2*, *LAT1*, and *LAT4* mRNA were relatively highly enriched in D3 cultures (Figure 1). For Na^+^-dep AATs, expression of *B^0^AT2 was* 21x, 3.7x and 7.5x more abundant than *ASCT2*, *y^+^LAT1*, *y^+^LAT2*, respectively. Among Na^+^-indep AATs, *LAT1* was expressed by D3 at 340x higher levels than *b*^0,+^*AT*, while *LAT4* was 115x than *b*^0,+^*AT* levels. Therefore, we considered whether the highly expressed AATs (B^0^AT2, LAT1, LAT4_low_) alone could account for measured Leu uptake rates using a restricted model. The maximum uptake rates, *V_max,i_* for B^0^AT2, LAT1 and LAT4_low_ were determined in the restricted model as 0.530 ± 0.612, 0.127 ± 0.136, and 6.97 ± 0.544 pmol/min respectively (reduced Chi^2^ =10^−9^, adjusted R^2^ = 0.986). Notably, neither error nor goodness of fit was improved for the former model that reported activity by y^+^LAT1 in addition to B^0^AT2, LAT1 and LAT4_low_ vs. the more restrictive model. In summary, considering potential activity by 8 AATs transports, the model calculated activity by two Na^+^-indep AATs, LAT1 and LAT4_low_, and two Na^+^-dep transporters, B^0^AT2, y^+^LAT1, were sufficient to carry out Leu transport in D3.

### Both sustained and acute growth factor supplementation impact hCMEC/D3 leu transport

Several protocols for D3 culture have been recommended. Initially, a “rich” medium (RM) supplemented with the growth factors basic fibroblast growth factor (bFGF), epidermal growth factor (EGF), vascular endothelial growth factor (VEGF) and insulin-like growth factor 1 (IGF1) was suggested to promote cell growth.^6^ Subsequently, a minimal media (DM) without EGF, VEGF and IGF1 was recommended to promote D3 differentiation rather than proliferation (personal communication Pierre-Olivier Couraud). We routinely used both media protocols and were interested in whether D3 cultured in the various media differed in Leu AAT activities and gene expression.

We compared total and fraction of Na^+^-dep Leu transport vs. days of D3 culture in RM vs DM. Total Leu uptake rates and percent Na^+^-dep uptake by D3 cells cultured in RM increased linearly for approximately 5 days and plateaued during the 2^nd^ week of culture (Figure S2(a)). Leu uptake by D3 cultured in DM fluctuated from ∼60–100% of cells grown in RM (Figure S2(b)). The fraction of Na^+^-dep Leu uptake by D3 grown in either media peaked at ∼5 days, then decreased (Figure S2(b)).The data are consistent with D3 exhibiting both Na^+^-dep and Na^+^-indep Leu uptake although contributions of Na^+^-indep AATs dominated at all phases of D3 culture.

**Figure 3.**
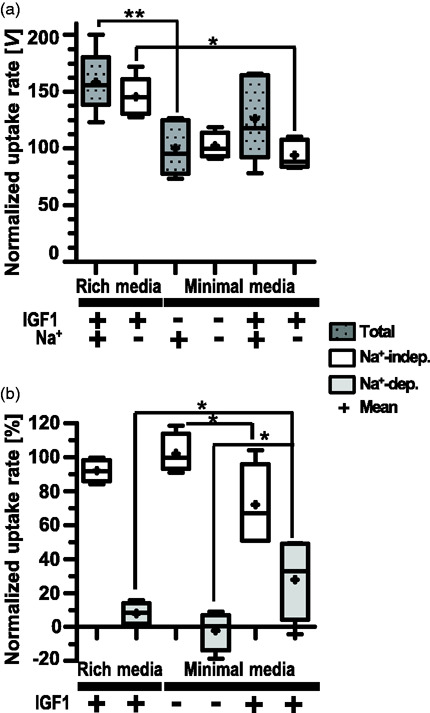
Effect of IGF1 on hCMEC/D3 on transport activity. 100 µM Leu uptake by D3 (p31–33) seeded at 4.5 × 10^4^ cells/cm^2^ and cultured for 2 days in rich medium vs. minimal medium (DM) with and without IGF1 supplementation as indicated. Leu uptake rates were normalized to uptake rates in the presence of Na^+^ by cells grown in DM without IGF1 supplementation. Panel a shows normalized total uptake vs Na-independent uptake (Na^+^-indep) rates [V] and panel b shown normalized percent [%] Leu uptake by Na-independent and Na^+^-dependent (Na^+^-dep) AATs. Data are plotted as box (25 to 75 percentiles) and whisker (minimum to maximum; line indicates median and + marks mean values) from 5–6 filters from two independent experiments. Asterisks indicate statistical significance as: p < 0.05 (*) and p < 0.01 (**).

D3 cultured for 2 days in RM prior to culture in DM for an additional 3 days (5d total culture) exhibited equivalent Leu rate and Na^+^-dep transport as cells cultured for 5d in RM (Figure S2(b)), suggesting the importance during the initial growth phase of growth factor supplementation for promoting AA transport. The insulin-like growth factor 1 (IGF1), which is known to be both transported across BBB^26^ and synthesized in the CNS,^27^ promotes cellular proliferation via the Akt/mTOR signaling pathways.^28^ We investigated potential early effects (day 2) of IGF1 in promoting Na^+^-dep transport. Figure 3(a) and (b) show 100 µM Leu uptake in ±Na^+^ by D3 cultured in RM vs. DM ± IGF1. Total Leu uptake was significantly higher for cultures grown in RM than in DM-IGF1. Addition of IGF1 to DM restored total uptake levels (Figure 3(a)). All detectable Leu uptake by cells grown in DM was due to Na^+^-indep transport, whereas cells grown in RM additionally had low (8%) but detectable Na^+^-dep AAT activity (Figure 3(b)). Addition of IGF1 to DM significantly increased the percent Na^+^-dep uptake (to 28% of total uptake) (Figure 3(b)).

### hCMEC/D3 genes show variable responsiveness to length of culture and/or IGF1

**Figure 4.**
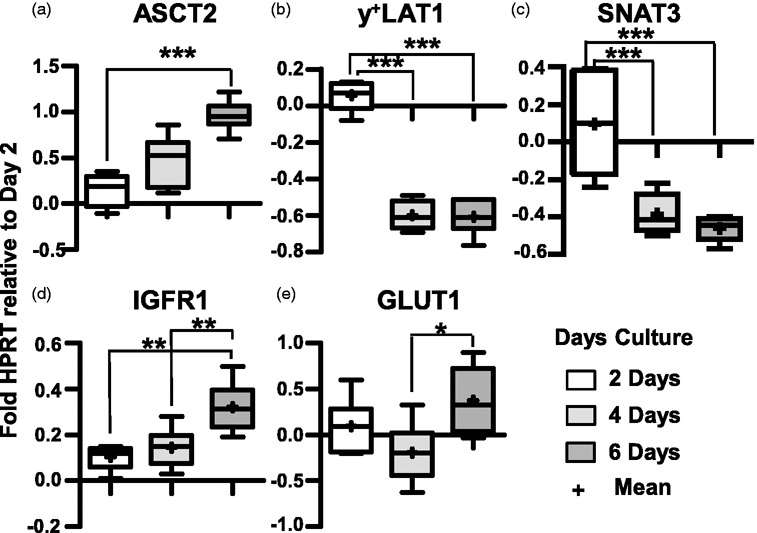
Effect of days of culture hCMEC/D3 gene expression. D3 mRNA expression of 2, 4, 6 day cultures relative to HPRT normalized to levels at 2 days of culture (2^−ΔΔCT^). Box (25 to 75 percentile) and whisker plots show minimum to maximum values with both median (line) and mean (+) values indicated. n = 6 filters (each sample value is the mean of 3 technical assays). Asterisks indicate statistical significance as: p < 0.05 (*), p < 0.01 (**), p < 0.001 (***) respectively.

**Figure 5.**
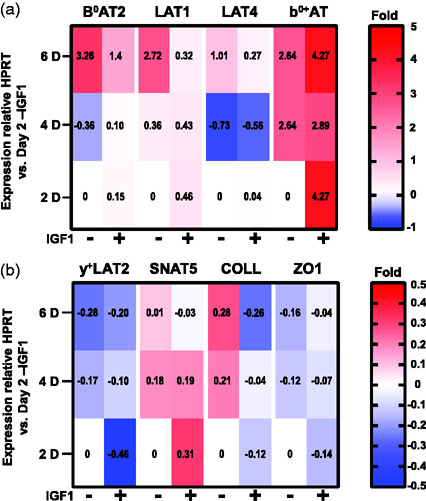
Effect of culture media and days of culture on hCMEC/D3 gene expression. Heat map plots of mean D3 gene expression changes by 2, 4, 6 day D3 cultured in DM ± IGF1 (mean values are reported in each cell). mRNA levels are relative to HPRT normalized to levels at 2 days in DM-IGF1 (2^−ΔΔC_T_^). (a) B^0^AT2, LAT1, LAT4, and b^0,+^AT mRNA expressions show up to 6x changes in expression. (b) y^+^LAT2, SNAT3, COLL and ZO1 show expression changes of up to 0.5x. For both panels, n = 3 biological replicates per condition as determined by 3 technical replicate assays for each sample.

We then tested effects of days of culture in DM ± IGF1 on D3 gene expression for 2, 4, 6 days culture in DM ± IGF1 relative to HPRT expression and normalized to expression at day 2 in DM (ΔΔC_T_ method). There were two categories of expression changes, namely those genes whose expression responded length of culture independent of IGF1 (Figure 4), and those influenced by both culture duration and IGF1 (Figure 5). No mRNA levels were significantly altered by IGF1 alone independent of length of culture. For most genes, expression differences were minor. Of the 17 genes tested, only five genes (*b^0+^AT*, *LAT1*, *LAT4, B^0^AT2*, *ASCT2*) showed at least a 2-fold change in expression from day 2 to 6 (Figures 4 and 5). D3 gene expression of four genes tested, ASCT1, SNAT1, SNAT2, and mTOR remained at approximately day 2 levels relative to HPRT regardless of culture time and media condition (data not shown).

For five genes transcription varied due to both ±IGF1 and length of culture. Surprisingly, for the three genes found most highly expressed by D3 in RM (*LAT1, B^0^AT2, LAT4,* Figure 1), supplementation of DM with IGF1 had an inhibitory effect (Figure 5(a)). *B^0^AT2* expression increased approximately 3X by day 6 in the absence of IGF1, whereas IGF1 treatment correlated with diminished increases to 2X baseline. Likewise, in the absence of IGF1, expression of *LAT1* increased nearly 3X by day 6, while IGF1 supplementation correlated with day 6 levels remaining at day 2 baseline. A similar effect of IGF1 was observed for *LAT4,* where expression, which dipped at day 4 before doubling relative to baseline at day 6 in the absence of IGF1, and only recovered to baseline levels in the presence of IGF1. In contrast, for *b^0+^AT* addition of IGF1 correlated with immediate and sustained increased expression as early as day 2. While, a complex relationship between growth factor supplementation, days of culture and D3 gene expression was observed, the data indicate that for most genes mRNA levels remained relatively constant.

## Discussion

Using integrated experimental and computational approaches, we interrogated the activity of individual AAT species in transporting LNAAs in the BBB employing the D3 cell line as a model system. Due to the differing kinetics and mechanisms by which AATs function, quantifying contributions of specific AATs mediating Leu, and by extension other solute transports, is key for using D3 to probe regulation of solute homeostasis. Importantly, given prerequisite conditions as we previously described,^14^ the procedure used in this study can be generalized to other cellular model systems. An advantage of this approach is in streamlining the process of assigning transporter species contributions to a targeted activity. Activities are calculated using the known MM kinetics of candidates generated from gene expression profiles independent of determination of protein expression and/or posttranslational state.

In this study, guided by a published set of microarray data,^15^ we first demonstrated that D3 grown in a supplement rich media express high mRNA levels for the AATs *B^0^AT2*, *LAT1*, *SNAT1*, *SNAT2* and *LAT4*, as well as for the signaling proteins *IGFR1* and *mTOR*. mRNAs for other known brain microvascular endothelial proteins such as *CLDN5*, *GLUT1* and *SNAT3* were expressed at hundred-fold lower levels (Figure 1). We then quantified the relative activities of specific AAT species in D3 plasma membrane Leu transport using a computational method previously developed to parse contributions of individual enzymatic species among suites of potentially active SLCs.^14^ For D3 cells, activity of the Na^+^-indep AATs LAT1 (exchanger), and LAT4 (uniporter) were robustly calculated in all scenarios to contribute to Leu uptake. Additionally, calculations considering only the three most highly expressed AATs showed B^0^AT2 activity was sufficient to account for Na^+^-dep Leu transport. Quantification of the contributions by all 8 potentially expressed AAT kinetic activities calculated y^+^LAT1, in addition to B^0^AT2, participated in Na^+^-dep AAT transport (Figure 2(b) and (c), Table S2). While mRNA levels are not necessarily predictive of protein expression, the model calculations support the conclusion that the highly expressed AATs LAT1, LAT4 and B^0^AT2, and possibly the less highly expressed y^+^LAT1, contributed to Leu uptake by D3 cells.

### Implications for physiological microvascular endothelial L-leucine transport

In any discussion of the physiological role of brain microvascular endothelial cells, it is important to note one of their crucial functions is in regulation of transendothelial solute transport. To accomplish this, BBB endothelial cells, like kidney and intestine epithelium, form tight polarized membranes with unique sets of proteins expressed on luminal and basolateral faces.^3^ When cultured on Transwell™ filters, as described here, D3 do not form a tight barrier to the diffusion of small molecular weight molecules such as AAs.^29^ Moreover, although polarized expression by D3 of P-glycoprotein, a major BBB marker has been documented,^30^ it is not clear the extent to which D3 AAT membrane localization is polarized. Therefore, the assays described here did not distinguish between apical and basolateral Leu uptake.

The well-characterized *in vivo* membrane localization of LAT1 on both blood and brain facing endothelial membranes, and the known LAT1 activity in brain endothelial microvascular cells, underscore the finding that LAT1 is a key D3 Leu transporter.^2,3,10,14,19,20^ Additionally, D3 protein expression of LAT1 has been previously proven by proteomic analysis.^9,10^ In contrast, although LAT4 has been shown to be present *in vivo* at low levels in brain tissue, to our knowledge, LAT4 has not been reported expressed by BBB endothelial cells.^24,31^ Although, both a low and a high affinity Leu transport has been reported,^24^ other studies have not replicated this finding.^31^ Our calculations for D3 Leu transport indicated activity by only a low affinity Leu transport by LAT4. Global knockout of mouse LAT1 leads to early embryonic lethality,^32–34^ while the constitutive *Lat4 null* mouse dies within 10 days postnatal possibly due to profound malnutrition.^31^ On the other hand, the conditional knockout of intestinal LAT4 resulted in no major phenotype indicating the lethal defect is not necessarily solely due to an intestinal LAT4 deficiency.^35^

B^0^AT2 has been proposed as a candidate for the unidentified Na^+^-dep transporter for LNAAs, whose activity has been detected in abluminal BBB endothelium membranes.^36,37^ High B^0^AT2 expression has been demonstrated in cerebral cortex, hippocampus, cerebellum, midbrain, and olfactory bulb, localized to BBB astrocytes and neurons.^38,39^ While, crude cortical synaptosomes prepared from B^0^AT2 knockout mice showed a 40% reduction in Leu transport, mice are viable and fertile with no notable phenotype presumably due to compensatory functional redundancy.^38,40^ Intriguingly, D3 express mRNA for the AAT associated protein collectrin, albeit at 10 fold lower levels than for B^0^AT2 (Figure 1). In the kidney, collectrin acts to promote SLC6A19/B^0^AT1 membrane expression and activity.^41–44^ Collectrin also interacts with SLC6A18/B^0^AT3 (B^0^AT3) to both promote surface expression and catalytic activity.^43,45^ Using mutational studies, Fairweather and coworkers identified several residues in murine B^0^AT3 (mB^0^AT3), and their homologous residues in human B^0^AT1 (hB^0^AT1) that mediate collectrin interactions. For most of the mB^0^AT3 residues that interacted with collectrin (Q25V, R225T, N283D), the homologous hB^0^AT1 wild-type residues (Q40, R240, N298) are identical in hB^0^AT2 (Q69, R269, N328). Furthermore, in D3, collectrin gene expression, like that of B^0^AT2, decreased in the presence of IGF1 (Figure 5). These data are suggestive of the possibility that hB^0^AT2 interacts with collectrin in a similar manner as hB^0^AT1. However, there was one mB^0^AT3 mutation (D21N) that increased mB^0^AT3 surface expression in the absence of collectrin. For hB^0^AT2, unlike for hB^0^AT1, the wild-type residue at that position is Asn^45^ implying that mB^0^AT2 might be expressed on the plasma membrane regardless of collectrin co-expression. To our knowledge, no studies of potential B^0^AT2-collectrin interactions have been published to date.

The antiporter y^+^LAT1, which like LAT1 associates with the SLC3A2/4F2HC heavy chain glycoprotein, mediates Na^+^-dep uptake of neutral AAs coupled with the Na^+^-indep efflux of positively charged AAs (Arg, Lys).^46,47^ y^+^LAT1 activity was calculated to contribute significantly to D3 Leu uptake. However, *in vivo* expression of y^+^LAT1, while ubiquitous, is relatively low in brain. Rather y^+^LAT1 is preferentially expressed in kidney and intestinal epithelium, as well as in lung tissues and leucocytes.^48^ Human y^+^LAT1 is best known for the +50 mutations resulting in Lysinuric Protein Intolerance (LPI), a urea cycle and multisystem disease with variable expression and outcome.^49^ Global y^+^LAT1 knockout is perinatally lethal and pups suffer from intrauterine growth restriction hypothesized to be due to concurrent downregulation of liver IGF1 during gestation.^50^ LPI patients may suffer neurological consequences such as acute encephalopathy, seizures, and cognitive disability, which are thought due to hyperammonemia episodes arising from high protein ingestion and that respond to citrulline treatment.^49^ An inducible murine y^+^LAT1 knockout restricted to renal and intestinal tissues nonetheless shows persistent neurological deficits that likewise resolve with citrulline supplementation.^51^ L-citrulline uptake has been shown in a BBB cell model to be mediated by LAT1.^52^ Taken together, the data do not point to a major physiological role for human brain microvascular endothelial localized y^+^LAT1 in BBB Leu transport.

### Influence of culture conditions on hCMEC/D3 L-leucine transport

As a consequence of conditions unique to cell culture and/or D3 immortalization, there is the possibility that D3 expressions of B^0^AT2, y^+^LAT1, and LAT4 are altered relative to physiological brain microvascular endothelial expression. For example, a previous study by our group compared gene profiles from highly purified, rapidly isolated endothelial mRNA from *in vivo* mouse brain microvascular endothelial cells (MBMEVC) with those from brief (5 day) cultures of the same primary cells, as well as with an immortalized mouse endothelioma cell line (b.END5). By microarray, B^0^AT2 and LAT4 expression was below cutoff in all sample types. Compared to the the low *in vivo* expression of y^+^LAT1, its expression in b.END5was elevated ∼6X. In contrast, the relatively high LAT1 expression *in vivo* was downregulated ∼6X in primary cultures and ∼17X in b.END5. We proposed the observed profound changes in expression between intact BBB endothelial cells and cultured cells arose from novel metabolic pressures and concurrent loss of essential signals promoting differentiation.^2^

In the current D3 study, media composition and culture growth phase correlated with changes in the expression of a number of genes. During the initial establishment of D3 cultures (∼1–5 days), the time course of increases in total transport were relatively comparable regardless of growth media (Figure S2). However, for cells cultured in DM, early supplementation with IGF1 correlated with a slight but significant boost in the fraction of Na^+^-dep uptake (Figure 3). Surprisingly, the gene expression of B^0^AT2, LAT1, and LAT4 decreased in the presence of IGF1 especially with increasing duration of culture. Conversely, y^+^LAT1 mRNA levels decreased with length of culture regardless of IGF1 (Figure 4). Confluent monolayers shed Na^+^-dep uptake somewhat more rapidly than overall transporter activity with increased time of culture (Figure S2). This suggests as D3 differentiate (and possibly, for *in vivo* differentiated BBB endothelial cells), LNAA transport relies on Na^+^-indep facilitated diffusion and obligatory exchanger activities in preference to Na^+^-dep concentrative AATs. This is reasonable, especially considering the steep gradient of Leu concentration between plasma and ISF that favors AA influx from blood to brain.^1^ In addition, it is consistent with previous data from our group demonstrating LAT1 rather than the Na^+^-dep SNAT1 and SNAT2 AATs controlled ISF influx of L-glutamine (Gln) from plasma.^1^

Taken together our results indicate LAT1, LAT4 and B^0^AT2 and possibly y*^+^* LAT1 mediate D3 plasma membrane Leu transport. However, while D3 recapitulate the physiological primacy of LAT1 for Leu transport; the evidence supporting translating results for LAT4, B^0^AT2, and y^+^LAT1, from D3 to human BBB endothelium is less compelling. Nonetheless, as has previously been postulated for epithelial tissues,^53–55^ potentially symporter, antiporter and facilitative AAT species functionally cooperate in modulating *in vivo* BBB LNAA transport and CNS AA homeostasis.

## Supplemental Material

sj-pdf-1-jcb-10.1177_0271678X211039593 - Supplemental material for Analysis of L-leucine amino acid transporter species activity and gene expression by human blood brain barrier hCMEC/D3 model reveal potential LAT1, LAT4, B^0^AT2 and y^+^LAT1 functional cooperation Click here for additional data file. Supplemental material, sj-pdf-1-jcb-10.1177_0271678X211039593 for Analysis of L-leucine amino acid transporter species activity and gene expression by human blood brain barrier hCMEC/D3 model reveal potential LAT1, LAT4, B^0^AT2 and y^+^LAT1 functional cooperation by Mehdi Taslimifar, Martin Faltys, Vartan Kurtcuoglu, François Verrey and Victoria Makrides in Journal of Cerebral Blood Flow & Metabolism
