# Supplementary material for: Analysis of L-leucine amino acid transporter species activity and gene expression by human blood brain barrier hCMEC/D3 model reveal potential LAT1, LAT4, B0AT2 and y+LAT1 functional cooperation
Source: J Cereb Blood Flow Metab. 2021 Aug 24;42(1):90–103. doi: 10.1177/0271678X211039593 (PMC8721536; doi:10.1177/0271678X211039593)

Analysis of L-Leucine Amino Acid Transporter Species Activity and Gene Expression by Human Blood Brain Barrier hCMEC/D3 Cell Model Reveal Potential LAT1, LAT4, B^0AT2 and y^+LAT1 Functional Cooperation.

Mehdi Taslimifar[1,2]*, Martin Faltys[2,3]*, Vartan Kurtcuoglu[1,4], François Verrey[2,4], Victoria Makrides[1,2,5#]

[1]The Interface Group, Institute of Physiology, University of Zürich, Zürich, Switzerland; [2]Epithelial Transport Group, Institute of Physiology, University of Zürich, Zürich, Switzerland; [3]Department of Intensive Care Medicine, University Hospital, University of Bern, Bern, Switzerland; [4]National Center of Competence in Research, Kidney.CH, Switzerland; [5]EIC BioMedical Labs, Norwood MA, USA; *These authors contributed equally to this work.

Running headline: Expression & activity of human BBB AATs

#Corresponding Author: Victoria Makrides

Interface Group, Institute of Physiology,
University of Zurich, Zurich, 8057-CH

Phone: +1.857.204.2474, +1.781.769.9450
Fax +1.781. 769.2099

makrides@access.uzh.ch,
vmakrides@eiclabs.com

Supplementary Information

Table S1: Human qPCR primers for SYBR Green listing NCBI Accession number, 5' to 3' sequence for forward and reverse primers and amplicon length.

Gene	Type	NM#	Forward primer	Reverse primer	Amp
HPRT	Reference	NM_000194	TTA TTG AGA GGA AAC GTC TTG	CCA GCA GGT CAG CAA AGA ATT	113
CLTRN	Associate	NM_020665.4	ACC TCT TCA AAG CGA TGG TAG CT	ACC CTC TGG GTT ACA TTG CAA	100
ASCT1	SLC1A4	NM_003038	GCA ACC GAT TAT AAA GTC GTC ACC	CAC TCC TAA CAC CAG AGC AAA CA	132
ASCT2	SLC1A5	NM_05628	CCT GGA TCT GAG AAA TAT CTT	CCT GGT TCC GGT GAT AAT CCT	134
B⁰AT2	SLC6A15	NM_182767	CTT TTC AGC AAC CCC TGC CT	TAG GTG GTG GCA GAA CTT TGT	79
LAT1	SLC7A5	NM_003486	GAA GGC ACC AAA CTG GAT GTG	TTG ATC ATT TCC TCT GTG ACG AA	107
y⁺LAT1	SLC7A7	NM_003982	GAG CTGCTT CGC CCC TTA T	CAG GGT TCC CCA TTT GAC AT	100
y⁺LAT2	SLC7A6	NM_003983	ACA CGT TCA CTT ACG CCA AGG	CCT CAA AGG CGT CCT GAA A	105
b^{0,+}AT	SLC7A9	NM_014270	TGA TGA CTG CCA CCG AAC TC	TAG CAG CAG CGA TGG TTG AA	103
LAT4	SLC43A2	NM_152346	CGA GGA GAA AGA CGC CAA CC	GAA GGC CCG CAT GGC ATTAG	88
SNAT1	SLC38A1	NM_030674	TAC CGT GGT TAC CTG CAT ACT CTT	GTT AGC AGA TGT AAC TCC TAC GAC TCC	103
LAT4	SLC43A2	NM_152346	AAGTAT GGC CCG AGC AAG CT	ACG GAG AGA GCG TTT GGT TTA C	101
SNAT2	SLC38A2	NM_018976	GGG ATA TAA GGC ATT TGG ATT AGT T	TTT ACA TAT GAA GAG GTA GCT TGA CA	100
SNAT3	SLC38A3	NM_006841	TTG GGA CCC CAG GAA AGC	AGT GGC AGC TCA GAC TTG ATG A	100
SNAT3	SLC38A5	NM_033518	CCC AGC CAA GGA CTC AAC A	CTC AGG GTG GCA GAC AAA AG	105
GLUT1	SLC2A1	NM_006516	CGG GCC AAG AGT GTG CTA AA	CTT CTTCTC CCG CAT CAT CTG	102
CLDN5	Tight junction	NM_001130861	GCC TTC CTG GAC CAC AAC A	GTC GTA CAC TTT GCA GTG CAT GT	105

OCLDN	Tight junction	NM_002538.2	TAT AAA TCC ACG CCG GTT CCT	CAA AGT TAC CAC CGC TGC TGT A	100
ZO1	Tight junction	NM_003257 NM_175610	TACAAT GGA AAA CTG GGC TCT TG	GCT GCT CAG CTC TGT TCT TAT TAG G	100
IGF1R	Receptor	NM_000875	CAA GTT GAG CAG CGA GAA TG	TGA ATC CGG GCT GTG TAG TTC	104
mTOR	Ser/Thr Kinase	NM_004958	GGC TGA TGG ACA CAA ATA CCA A	TGG TCC CCG TTT TCT TAT GG	132

Table S2: OriginPro model description (versions 2020, 2021; Origin Lab, Northampton, MA, USA.

**Iteration Algorithm**	Levenberg Marquardt							
**Equation**	y1=x*V1/(x+0.367)+x*V2/(x+0.16)+x*V3/(x+0.032)+x*V4/(x+0.236) +x*V5/(x+0.0317)+x*V6/(x+0.103)+x*V7/(x+3.733)+x*V8/(x+1.1);<br>y2=x*V3/(x+0.032)+x*V6/(x+0.103)+x*V7/(x+3.733)+x*V8/(x+1.1);							
**Parameters**	V1	V2	V3	V4	V5	V6	V7	V8
**Parameter Meaning ($V_{max,i}$)**	ASCT2	B^{0}AT2	LAT1	y$^+$LAT2	y$^+$LAT1	LAT4h$_{igh}$	LAT4$_{low}$	b$^{0,+}$AT
**Calculated Values ($V_{max,i}$) pM/min**	0	0.2492±15	0.0927±0.4	0	0.2161±0.7	0	7.448±8.95	0

Figure S1: Time course of hCMEC/D3 L-Leucine uptake. hCMEC/D3 (D3) cells were plated on plastic collagen coated tissue culture wells in rich media (EBM2 supplemented with growth factors) for 5-6 days. Uptakes (nmol/well) are shown as mean ± SD were carried out in Na^+ containing solution using for various times (1, 3, 5, 10, 15, 20, 30 min). n = 6 from 2 independent experiments.

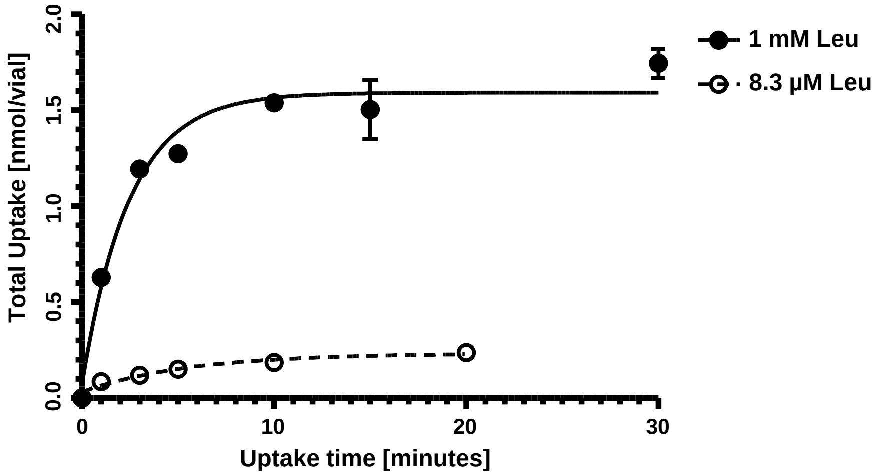

Figure S2. L-leucine transport activity in hCMEC/D3 (D3) cells cultured on Transwell™ filters for various lengths of time in rich (RM) or minimal (DM) media. **a**) hCMEC/D3 (p31-37) were plated on rat-tail 1 collagen coated Transwell™ filters at 4.5 X 10^4 cells/cm^2 and cultured in PM for 2, 4, 5, 7, 10, 13, 14 days . Total L-leucine (Leu) uptake (50 µM Leu except day 2 data, which is for 100 µM Leu) in sodium (Na^+) solution was reported as mean ± SD [pmol/(min·cm^2)]. **b**) D3 were plated on rat-tail 1 collagen coated Transwells™ as in panel **a** in RM and maintained in RM for 1, 2, or 7 days followed by culture in DM for 3, 5, 8 or 7 days as indicated. Total uptake rate [pmol/(min*cm2)] of 50 µM Leu in Na^+ is expressed as a fraction of total Leu uptake rate [%] by D3 cells cultured in PM for the same experiment for the indicated total days of 4, 5, 7, 10, and 14. Na^+-dep Leu uptake is shown, respectively, for both cells cultured as indicated in PM/DM or PM alone. n = 2 - 3 filters from 8 experiments. Data are shown as box (25 to 75 percentiles) and whisker plots of minimum to maximum with median (line) and mean (+) values indicated.

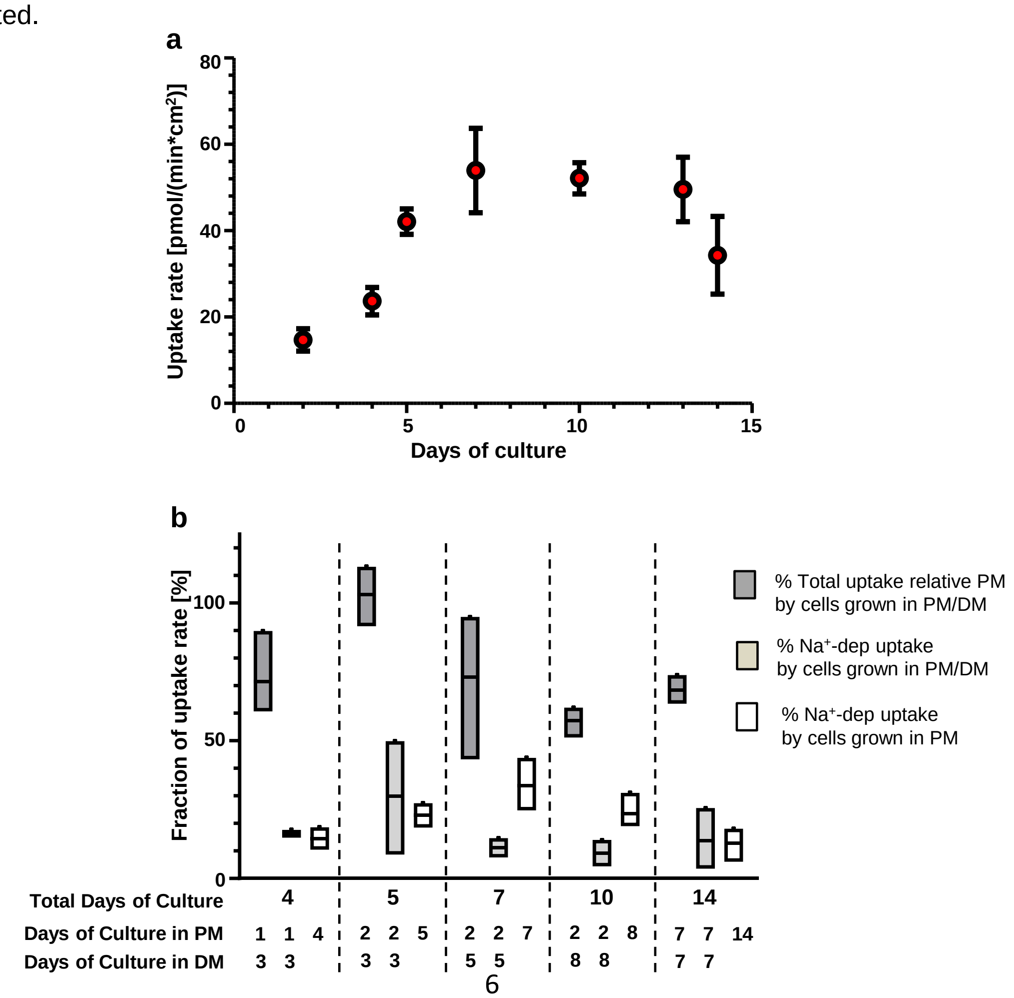

Supplement: sj-pdf-1-jcb-10.1177_0271678X211039593 - Supplemental material for Analysis of L-leucine amino acid transporter species activity and gene expression by human blood brain barrier hCMEC/D3 model reveal potential LAT1, LAT4, B0AT2 and y+LAT1 functional cooperation [file sj-pdf-1-jcb-10.1177_0271678X211039593.pdf]
